# MIESRA mHealth: Marital satisfaction during pregnancy

**DOI:** 10.1371/journal.pone.0289061

**Published:** 2023-08-24

**Authors:** Besral Besral, Misrawati Misrawati, Yati Afiyanti, Raden Irawati Ismail, Hidayat Arifin

**Affiliations:** 1 Department of Biostatistics, Faculty of Public Health, Universitas Indonesia, Depok, Indonesia; 2 Department of Maternity and Women Health, Faculty of Nursing, Universitas Indonesia, Depok, Indonesia; 3 Department of Maternity and Women Health, Faculty of Nursing, Universitas Riau, Pekanbaru, Indonesia; 4 Department of Psychiatrics, Faculty of Medicine, Universitas Indonesia, Depok, Indonesia; 5 Department of Fundamental Nursing Care, Faculty of Nursing, Universitas Airlangga, Surabaya, Indonesia; 6 School of Nursing, College of Nursing, Taipei Medical University, Taipei, Taiwan; University of Rome La Sapienza: Universita degli Studi di Roma La Sapienza, ITALY

## Abstract

The transition of a pregnant woman’s role often causes emotional changes that have an impact on marital satisfaction. We develop MIESRA mHealth and evaluate its impact on satisfaction of husband-wife relationship during pregnancy. A quasi-experimental study was conducted on 82 couples of pregnant women and divided into control, single, and paired group. We implemented MIESRA mHealth for four weeks. In the couple group, the wife did mindfulness based on the information in the MIESRA mHealth together with her husband. In a single group, the wife sees the video as an initial guide to doing mindfulness. In the control group, respondents received programme interventions from hospitals which included education and consultation with obstetricians. Husband-wife relationship is evaluated using Compatibility of Husband-and-Wife Relationships / *Kesesuaian Hubungan Suami Istri* (KHSI) questionnaire and the generalised estimating equations (GEE) was used to analyse the data. The women’s KHSI scores in the couple and single intervention groups (β = -7.46, p = 0.002; β = -9.11, p = 0.001) were better than the control group. The husbands’ KHSI scores in the paired and individual intervention groups (β = -7.04, p<0.001; β = -3.74, p = 0.024) were better than the control group. Nursing interventions to build emotional bonds between parents and foetuses based on mHealth can be a promising intervention for marital harmony during the perinatal period. MIESRA m-Health is a promising intervention on marital satisfaction during pregnancy and can be implemented as a part of the antenatal care programme to increase marital satisfaction.

## Introduction

Pregnancy is a transition period in which women strive with biochemical, physiological and anatomical alterations [1]. Transition to motherhood is influenced by various factors across different levels, including individual factors (partner support, career aspirations), organizational factors (family-friendly work practices, role models), and societal factors (social norms, attitudes towards the maternal body) [2]. Some pregnant women adjust well to these changes by maintaining optimal physical condition, positive interpersonal relationships, and gaining support from their family and health workers [3–5]. However, the transition is sometimes beyond control and makes pregnant women vulnerable to physical, mental, and social illnesses [6, 7]. Pregnant women mostly face psychological issues such as anxiety and depression during transition to motherhood [8, 9]. This condition can have an impact on miscarriage, or the baby is born with stunting and both mental and physical disabilities [10, 11]. Other conditions are such as abortion, premature delivery, pre-eclampsia, placental abruption, and lack of concern for the foetus [12–15]. Not only does it have an impact on the growth and development of the foetus, but also this condition has an impact on the condition of marital satisfaction, which leads to miscommunication and disharmony [16, 17]. The previous study mentioned a relationship between the foetus and the transition to parenthood during pregnancy [18, 19]. The exceptional relationships between pregnant women, their husbands, and foetuses are placed at the core of the living environment that needs to be strengthened to maintain their wellness [20, 21].

The couple’s orientation to everyday life, as well as changes to daily life, joyfulness experienced, and their subsequent adaptation to those changes, is reflected in their marital satisfaction [22]. Couples who can talk and work out their differences on key marital issues to the satisfaction of both partners have a harmonious marriage [23, 24]. During pregnancy, marital satisfaction plays an important role in maternal psychology, foetal development, and also the coverage of vaccination for baby well-being [25–28]. To reach this, husbands should understand the emotional condition of pregnant women caused by hormonal fluctuations [29]. Becoming a mother theory from Mercer [30] states that, at the stages of commitment, attachment and preparation, it can increase the bond between mother and children as well as environmental factors that also have an impact on husband- wife marital satisfaction.

To increase the closeness of the relationship between parents and foetus as well as to increase marital harmony, mindfulness interventions can be used as a reference. These interventions can reduce psychological distress and improve emotion regulation and empathy [31]. Previous study mentioned high correlation between mindfulness and marital satisfaction [32]. Other studies about mindfulness that that specific on childbirth and parenting have a positive improvement in mother’s wellbeing and parents’ relationship [33, 34]. Mindful technique helps both husband and wife to acknowledge any perceptions, problems, and recognise any disadvantages and advantages in their marital life. This technique also helps to identify the positive perception and satisfying marital relationship by creating open-minded discussion to solve any problems, including pregnancy condition [35, 36].

To date, research indicates that the utilisation of mobile health (m-health) in providing nursing interventions for pregnant women is possible [37]. Technically, mindfulness intervention that emphasises on maternal relationship is implemented manually and online [38–40]. Thus, we developed mHealth-based mindfulness namely “Fostering the Emotional Bond of Parents and Foetus/*Menjalin Ikatan Emosional Orangtua dan Janin* (MIESRA)” to facilitate pregnant women and husbands that can be implemented everywhere and anytime to maintain the marital life during pregnancy for mothers’ and foetus wellbeing. MIESRA mHealth provides many interventions to help the mother maintain the pregnancy and foetal condition and marital satisfaction for both husband and wife. This mHealth can be used by both husband and wife. Thus, we conducted this study with aim to assess the effect of MIESRA as a mobile health-based nursing intervention to increase the satisfaction in a husband-wife relationship.

## Materials and methods

### Study design

We utilised a quasi-experimental study to evaluate the effectiveness of MIESRA mHealth to the outcome variable [41, 42]. This study design is appropriate since the author did not perform randomisation. We faced limitation on study settings and subjects’ recruitment during the COVID-19 pandemic in Indonesia.

### Setting and sample

**Fig 1:**
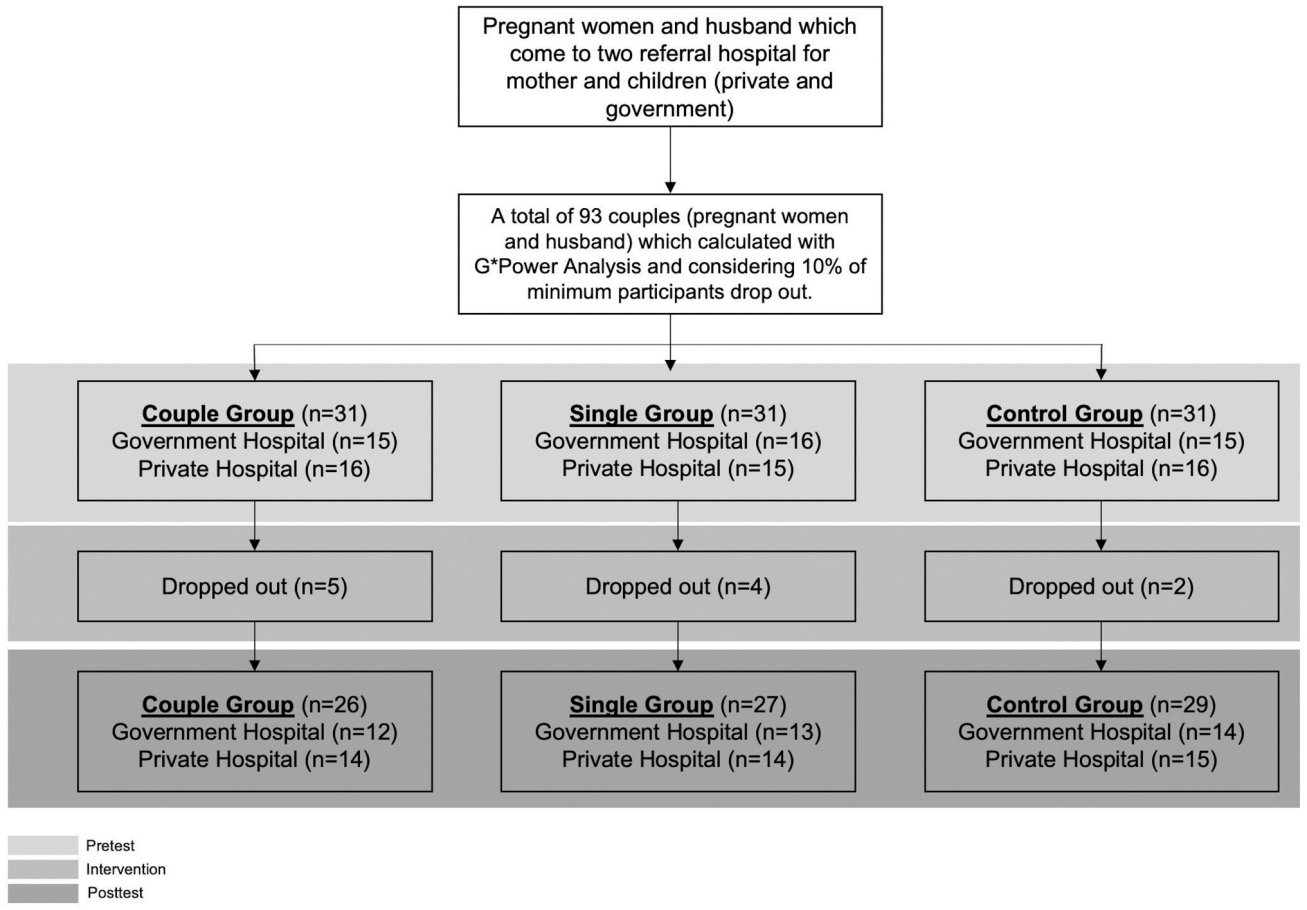
Sample diagram flow chart.

The study is conducted in the government and private hospitals in Jakarta, Indonesia. These hospitals were elected as a referral mother and children hospital in Indonesia. For homogeneity reasons, the data collection for all groups was implemented in both hospitals (Fig 1). We performed the study from baseline to three months (May to July 2021).

A total of 93 couples (husband and wife) participated in this study. A purposive sampling technique was used to recruit the participants. A total sample was estimated of 10% dropping out from this study. We used G*Power (software used to calculate statistical power) version 3.1.9.6 with the assumption of p < 0.05, 0.9 of power estimation, and 0.8 of effect size [43, 44]. Furthermore, we divided into three groups, namely couple groups, single groups, and control groups. Each group consisted of 31 couples. The inclusion criteria consisted of 1) pregnant women with *primigravida* or *multipara*; 2) a minimum of secondary education level; 3) gestational age between 20 and 34 weeks; 4) pregnant women aged 20–45 years; 5) husband willing to participate; 6) couple has smartphone with Android-based system; 7) never diagnosed with or under medication for mental health problems. In addition, couples who drop out during the study process will be excluded. At the end, 11 couples e dropped out (two couples resigned during the study process and nine mothers were delivered before the post-test data were collected). Finally, a total of 82 couples was included (Fig 1).

### Instruments

We used Compatibility of Husband-and-Wife Relationships / *Kesesuaian Hubungan Suami Istri* (KHSI) questionnaire to observe marital satisfaction [45]. This questionnaire was used by the author as a consideration with participants’ characteristics in Indonesia. This instrument consists of 29 items that cover six components 1) closeness; 2) conformity; 3) understanding; 4) a reflection of affection; 5) satisfaction; and 6) a husband-wife togetherness. In favourable statements, the score starts from 0 to 3 (items no. 1–14, 16,19, 20, 23–29). In the negative statements, the score starts from 3 to 0 (items no. 15, 17, 18, 21, 22). A lower score indicated satisfied and high score indicated not satisfied. A mean cut-off point was used to interpret the score of KHSI. The value of validity and the internal confidence consistency had a Cronbach’s alpha of 0.86. A reliability test of KHSI was conducted on 116 pregnant women in urban areas with Cronbach’s alpha of 0.89 [46].

#### MIESRA mHealth

MIESRA mHealth is an interactive learning media for pregnant women and their husbands that can guide how to build emotional bonds between parents and foetuses. This application is built using Android Studio version 4.1.2 which can be installed on devices such as mobile phones with Android Operating System 4.0 or the latest. This application consists of two types, namely applications for wives and applications for husbands and can be downloaded from https://miesra-app.nfcworld.web.id/. Detailed information can be seen in Table 1 & S1 Data. MIESRA mHealth has been tested through several steps. 1) At the beginning, MIESRA was tested to five users to evaluate the menus and function in detail. 2) The second steps, a limited test to five couples (pregnant women and husband), was used to evaluate the applicability and effectivity of application to reach the purpose. 3) The next step is the evaluation on efficacy, efficiency, and usability using Heuristic Evaluation (HE) approach [47]. HE was performed by an expert in nursing informatics and working as an analyst in the Digital Transformation Office at the Ministry of Health of Republic of Indonesia. Based on the HE assessment, MIESRA mHealth received 0.6 value (1 is the highest value) which means that MIESRA needed a minor revision and can be approved after revision. 5) In the last steps, a trial test used System Usability Scale (SUS) [48] to 15 couples in the government hospitals. Based on SUS test, MIESRA mHealth received 80.1 score, which means MIESRA is acceptable and can be used with the excellence category. We obtained Intellectual Property Rights approval from the Indonesian Ministry of Law and Human Rights (No. 000291880).

**Table 1:** MIESRA mHealth content information.

Indicator	Detail	Wife	Husband
Apps instruction	Contain a schematic of the steps to be carried out	✔	✔
Pretest and posttest	Contain three components of an assessment of the condition of pregnant women including psychological health, emotional bonding of mother and foetus and satisfaction of husband-wife relationship.	✔	✔
Daily activities	Consist of three components. 1) The video contains how to build an emotional bond between parents and their foetus through Mindfulness techniques, namely the technique of practicing thoughts, feelings and being aware of the condition of the pregnancy through interaction with the foetus, which is guided by a facilitator using audio media and a video dissertation on how to do it; 2) Mindfulness audio; and 3) Mother’s dairy notes.	✔	✖
Education	Contains health education materials on how to form emotional bonds consisting of 1) Pregnancy period (fetal development, physical changes in pregnant women, psychological changes, and how to bond during pregnancy between couples and with the foetus); 2) Period after childbirth (mother’s physical changes after childbirth, psychological changes, and how to bond during pregnancy between couples and with the baby).	✔	✔
Documentation	Consist of information about the result of pre and posttest value and suggestion to improve the health condition.	✔	✔

### Study procedures

**Fig 2:**
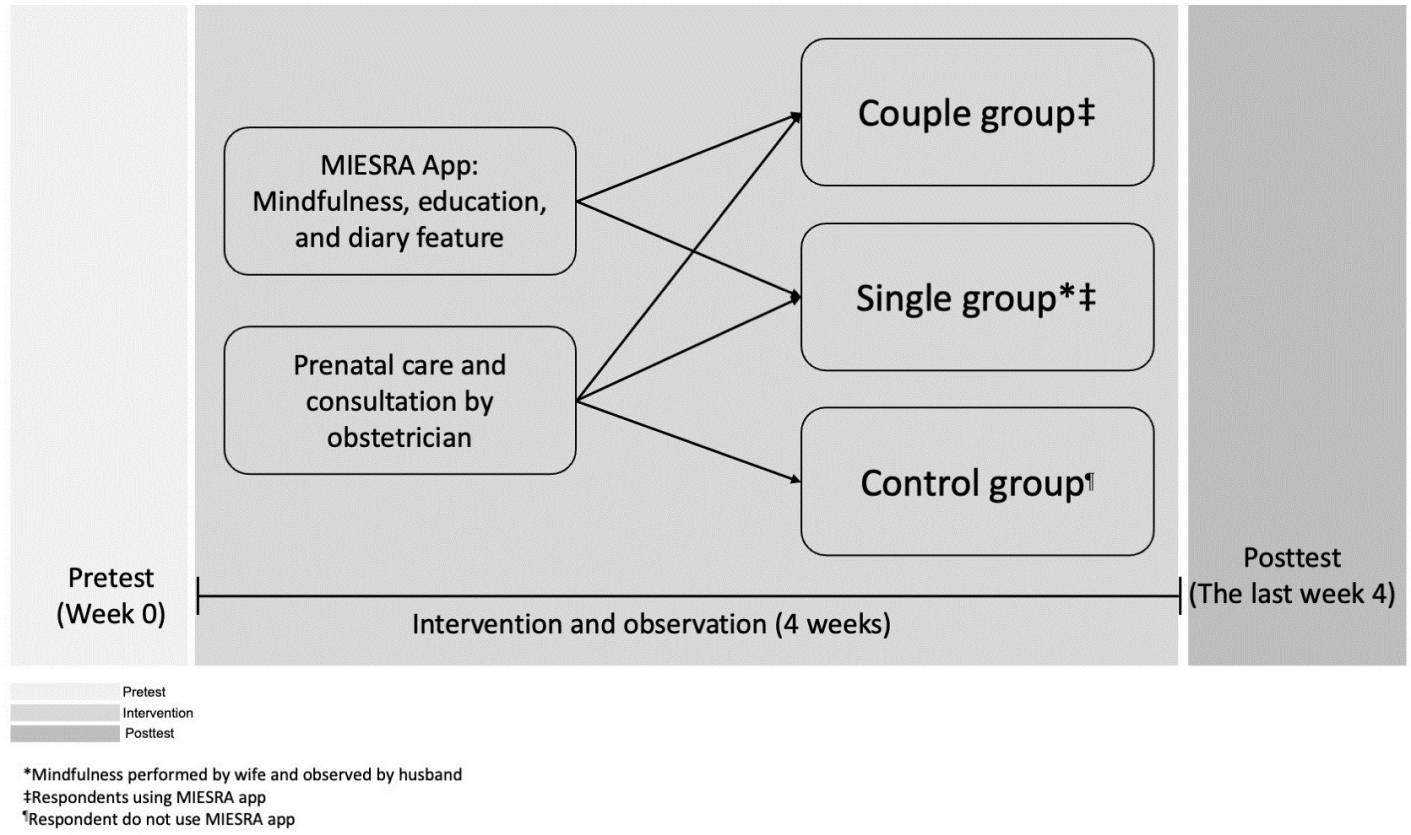
Study procedure diagram flow.

During the COVID-19 pandemic, we performed the study in two referral hospital in Jakarta, Indonesia. We set the study procedure with the condition of COVID-19 by following the Indonesia Government and hospital rules. We conducted the assessment from baseline to three months. At the beginning, we explained all the information about the study purpose and procedure to the respective respondents and asked them manually to sign the informed consent. Then, all the respondents in the couple and single groups were asked to download the MIESRA mHealth to their own mobile phone. All participants in the three groups received the same intervention in the form of prenatal care, education, and consultation from an obstetrician (See Fig 2). Respondents were allowed to consult with researchers via telephone or chat.

The pre-test was conducted to find out initial information regarding marital satisfaction through the MIESRA mHealth, except for the control group who used a special website provided by the researcher. Researchers could monitor all developments and information on the MIESRA mHealth through a special account.

In the couple group, the wife did mindfulness based on the information in the MIESRA mHealth together with her husband. This intervention was carried out based on the mindfulness guide available in the wife-MISRA mHealth. Husband and wife simultaneously viewed the video as an initial guide to mindfulness. Then, they were asked to listen to mindfulness-related audio from the wife-MIESRA mHealth and carry out the instructions with their husbands. This intervention was two times a week for four weeks with 15 minutes of each session. In addition, there is a Diary feature in the wife-MIESRA mHealth that helps the wife to write, submit complaints, and progress during the pregnancy process. This feature can be viewed and accessed by husbands (using the husband-MIESRA mHealth) with the aim to increase the husband’s knowledge regarding their wife’s condition during the pregnancy. Researchers monitor the activities carried out by couples through the application media and will remind them through the media in the app or by telephone if they do not intervene. Then, post-test was performed after four weeks of intervention.

In a single group, the wife sees the video as an initial guide to doing mindfulness. Then they listen to mindfulness-related audio and do it according to the instructions. In this group, the husband did not participate in mindfulness and only monitored his wife. This intervention was two times a week for four weeks with 15 minutes of each session. Then, the wife filled out a diary on the MIESRA mHealth feature to convey what she feels during pregnancy. In this group, husbands received education through the husband-MIESRA mHealth and could directly monitor the contents of the wife’s diary using the husband-MIESRA mHealth. Then post-test was done after four weeks of intervention.

In the control group, respondents received programme interventions from hospitals which included education and consultation with obstetricians. What makes this group different is that they didn’t get the MIESRA mHealth facility. Couples were allowed to ask researchers related to the research process via telephone or chat. Next, the post-test was conducted after four weeks and respondents received the MIESRA mHealth facility and an explanation of how to use it from the researcher.

### Data analysis

IBM SPSS version 24 was used to conduct statistical analysis. The descriptive characteristics of the respondents in all groups were represented using descriptive statistics such as numbers, percentages, and homogeneity test. Thus, one-way ANOVA test was utilised to determine the likelihood of homogeneity in all groups (homogeneity p = >0.05). Furthermore, we performed Generalized Estimating Equations (GEE) of multivariate analysis to evaluate the effectiveness of MIESRA mHealth. GEE is a development of the Generalized Linear Model (GLM) which is used to measure data that contain autocorrelation. The dependent variable can be in the form of numeric or categorical data and can control covariate variables that are dynamic or changing, whereas the GLM can only measure the numerical dependent variable and the data are static [49, 50]. In this study, the covariate contained a dynamic dependent variable because the pre-test value was different from the characteristic value, which was static. During the statistical analysis, we performed and consulted to the statistical expert in Universitas Indonesia.

### Ethical consideration

This research was ethically approved by the Human Research Ethics Committee of the Nursing Faculty, Universitas Indonesia (No. SK-243/UN2.F12.D1.2.1/ETIK. FIK. 2019) and the hospitals’ ethical committee. All respondents were over 18 years old, their privacy protected, and voluntarily authorised the written informed consent. The individual in this study has given written informed consent to publish this case detail. The individuals pictured in S1 Data have provided written informed consent (as outlined in the PLOS consent form) to publish their images alongside the manuscript, and the animated images presented in the S1 Data were drawn by the author.

## Results

From the total 82 couples included in this study, we present the demographic data in women and husband separately. We found that women are predominantly in the low risk aged from 20 to 35 years. Most of the women in the three groups completed college or university and have planned pregnancies. An almost similar number of women works outside home as with the homemaker ones. More women in the intervention groups live with their partners and children only rather than with extended family, but these are different with the control group. Overall, the characteristics among women in the groups shows homogeneity (p-value >.05), unless for the parity. In the husband category, the majority are young adults (age 26 to 35 years), had university education level, work as a private employee, and earn above the Jakarta’s regional minimum wage of IDR 4,200,000). Statistically, there are no differences in the characteristics of the father among the groups (p-value >0.05). Thus, the three groups in this study are homogenic (Table 2).

**Table 2:** Demographic and pregnancy characteristics of the respondents (n = 82 couples).

Demographic Characteristics	Control group (n = 29 couples)	Single group (n = 27 couples)	Paired group (n = 26 couples)	Homogeneity Test
	n (%)	n (%)	n (%)	p-value
Pregnant Women:				
Age				
• Low risk (20–35 years)	25 (86)	24 (89)	23 (88)	(0.90)
• High risk (<20 or >35 years)	4 (14)	3 (11)	3 (12)	
Last education				
• High school	8 (38)	7 (26)	8 (31)	(0.85)
• University/college	21 (72)	20 (74)	18 (69)	
Occupation				
• Homemaker	15 (52)	14 (52)	12 (46)	(0.80)
• Working outside home	14 (48)	13 (48)	14 (54)	
Parity				
• Primipara	16 (55)	11 (41)	18 (69)	(0.01)
• Multipara	13 (45)	16 (59)	8 (31)	
Pregnancy				
• Planned	21 (72)	14 (52)	15 (58)	(0.07)
• Unplanned	8 (28)	13 (48)	11 (42)	
Type of family				
• Nuclear	14 (48)	16 (59)	14 (54)	(0.51)
• Extended	15 (52)	11 (41)	12 (46)	
Husband:				
Age				
• Youths (17–25 year	2 (7)	1 (4)	3 (12)	(0.69)
• Young adults (26–35 year)	19 (65)	21 (78)	16 (62)	
• Middle-aged adults (36–45 year)	8 (28)	5 (19)	7 (27)	
Last education				
• High School	7 (24)	6 (22)	9 (35)	(0.55)
• University	22 (76)	21 (78)	17 (65)	
Occupation				
• Civil servants	3 (10)	5 (18)	5 (19)	(0.62)
• Private employee	19 (66)	20 (74)	17 (65)	
• Entrepreneur	4 (14)	2 (8)	2 (8)	
• Labor	3 (10)	0	2 (8)	
Income				
• High (< IDR 4,200,000)	22 (76)	22 (81)	18 (69)	(0.64)
• Low (≥ IDR 4,200,000)	7 (24)	5 (19)	8 (31)	

The homogeneity test of harmonious relationship score of the female and male groups before the intervention reported that the three groups (single, paired, and control) showed similar KHSI scores pre-intervention (p-value = 0.707 for women and p = 0.394 for men). Therefore, the differences that occurred post-intervention were influenced by the adoption of mindfulness exercises through MIESRA mHealth (Table 3).

**Table 3:** Husband and wife satisfaction score before and after MIESRA mHealth intervention (n = 82).

Variable	Control group (n = 29)			Individual Intervention (n = 27)			Paired Intervention (n = 26)			Homogeneity Pretest: F-score (p-value)
	Pretest	Posttest	Mean difference	Pretest	Posttest	Mean difference	Pretest	Posttest	Mean difference	
**Women Score**										
Mean	12.79	18.41	5.62	15.04	7.81	-7.23	15.38	8.27	-7.11	0.35
SD	8.21	10.91	(<0.001)	10.18	5.71	(<0.001)	11.12	5.1	(<0.001)	-0.707
**Husband’s Score**										
Mean	11.76	15.86	4.11	13.11	9.33	-3.78	13.35	6.308	-7.04	0.94
SD	1.61	1.3	-0.003	1.67	1.34	-0.006	1.7	1.37	-0.005	-0.394

It can be seen in Table 4 that there were two variables included in the women’s KHSI multivariate test (intervention variables and husband’s KHSI). Three variables were included in the husband’s multivariate test (intervention variables, husband’s age, and women’s KHSI).

**Table 4:** Candidate confounding variables in modeling the effects of MIESRA mHealth on satisfaction of husband-wife relationships.

	Women’s KHSI score		Husband’s KHSI score
Static Independent Variables	p-value	Static Independent Variables	p-value
Intervention	0.001*	Intervention	<0.001*
Age	0.818	Age	0.001*
Education	0.483	Education	0.707
Occupation	0.818	Occupation	0.661
Parity	0.522	Income	0.633
Pregnancy plan	0.775		
Family living together	0.671		
Abortion history	0.506		
Pregnancy problem	0.812		
**Dynamic Independent Variables**			
Husband’s KHSI	<0.001*	Women’s KHSI	<0.001*

*Significance <0.01

### MIESRA mHealth towards women’s satisfaction score

Both the complete model and the final model of the variables that affect the women’s KHSI score after the intervention were the provision of the intervention, time, and the husband’s KHSI with a significance value lower than 0.001. The final model showed the women’s KHSI score was 3.68 points better than before the intervention. The husband’s KHSI also affects the mother’s KHSI condition. After the intervention, the mother’s KHSI score in the paired intervention group was 7.46 points better than the control group. In the individual intervention group, the mother’s KHSI condition was 9.11 points better than the control group after the intervention (Table 5).

**Table 5:** Modeling effect of MIESRA mHealth on women’s satisfaction score.

Independent Variable	Full	Model	p	Final	Model	p
	Coef. B	95% CI		Coef. B	95% CI	
Intercept	7.13		0.004	7.23		<0.001
Intervention						
Paired	1.83	-2.70–6.36	0.427	1.84	-2.67–6.35	0.424
Individual	1.59	-2.61–5.78	0.459	1.6	-2.71–5.92	0.467
Control	*Ref*.			*Ref*.		
Measurement Time						
Posttest	-3.65	0.75–6.54	0.014	3.68	0.99–6.36	0.007
Pretest	*Ref*.			*Ref*.		
Husband’s KHSI	0.47	0.22–0.72	<0.001	0.47	0.22–0.73	<0.001
Intervention*Time						
Paired*Posttest	-7.34	-12.89 –(-1.86)	0.009	-7.46	-12.10 –(-2.83)	0.002
Individual*Posttest	-9.05	-13.33 –(-4.77)	<0.001	-9.11	-13.25 –(-4.98)	0.001
Control*Posttest	*Ref*.			*Ref*.		

0.0 = Reference

### MIESRA mHealth towards husband’s satisfaction score

Both the complete model and the final model of the variables affecting the KHSI score after the intervention are the provision of the intervention, time and the women’s KHSI with a value of significantly less than 0.001. The final model showed that the husband’s KHSI score was 2.29 points better than before the intervention. The mother’s KHSI also affects the husband’s KHSI condition. After the intervention, the husband’s KHSI score in the paired intervention group was 7.04 points better than the control group. In the individual group, after the intervention, the husband’s KHSI condition was 3.74 points better than the control group (Table 6).

**Table 6:** Modeling effect of MIESRA mHealth on husband’s satisfaction score.

Independent Variable	Full	Model	p	Final	Model	p
	Coef. B	95% CI		Coef. B	95% CI	
Intercept	10.41		0.044	7.64		<0.001
Intervention						
Paired	1.2	-2.79–5.19	0.555	1.75	-3.07–4.57	0.699
Individual	0.87	-2.90–4.63	0.652	0.63	-3.24–4.50	0.75
Control	*Ref*.			*Ref*.		
Measurement Time						
Posttest	1.98	0.53–3.42	0.007	2.29	0.86–3.73	0.002
Pretest	*Ref*.			*Ref*.		
Women’s KHSI	0.29	0.05–0.53	0.018	0.32	0.07–0.58	0.012
Intervention*time						
Paired*Posttest	-5.84	-9.16 –(-2.52)	<0.001	-7.04	-9.75 –(-4.32)	<0.001
Individual*Posttest	-2.29	-6.04–1.47	0.232	-3.74	-6.99 –(-0.50)	0.024
Control*Posttest	*Ref*.			*Ref*.		

## Discussion

Marital satisfaction during pregnancy is needed as a husband’s support system. This has an impact on the psychology of the mother and the development of the foetus. For this reason, the MIESRA mHealth was created to help increase marital satisfaction among couples during pregnancy by using a mindfulness approach. Pregnant women used the MIESRA mHealth to establish emotional bonds between parents and foetuses with partners or individually.

In our study, we found that respondents in the paired and single group showed a better result in KHSI score compared to control group. It means that the MIESRA mHealth has a positive effect on marital satisfaction. As we mentioned that the MIESRA mHealth was developed based on mindfulness approach, the finding is supported by previous study using Mindfulness-Based Childbirth and Parenting (MBCP) programme that also increases the marital satisfaction [33]. MIESRA mHealth increased communication and mutual understanding of partners as experienced during childbirth. Husband was fully present in helping from preparation for delivery to completion of labour by reminding and guiding the wife in practising mindfulness activities. This is similar to previous study which mentioned that mindfulness practice during pregnancy increased the interpersonal relationship and physical effect, such as relaxation and comfort with the husband [51].

The success of mindfulness practice in increasing the marital relationship does not have to be performed manually in the class. It can be assisted by mHealth as described in this current study. A previous study mentioned online mindfulness as new methods to be more applicable in an easy way [40]. Moreover, mindfulness practice resulted in quality marital satisfaction and psychological wellbeing [38, 39, 52]. Mindfulness exercises carried out in the early stages have shown significant results in improving the quality of husband-and-wife relationships [53]. Mindfulness can effect to marital satisfaction through psychoeducation, daily registration and assessment of daily negative experiences, and a short mindfulness practice (5–15 minutes per day) [35, 38]. Those steps are important in mindfulness to increase the understanding from both couple and lead to happy marital satisfaction and wellbeing in general.

The improvement in the father’s KHSI score in the paired intervention group is better than in the single group. Our finding shows that mindfulness practice interventions carried out jointly by married couples produce a better result than individual interventions. Therefore, we suggested that pregnant women prioritise mindfulness exercises with their husbands to achieve a maximum harmonious relationship. Previous studies mention that mindfulness performed with couples leads to effective communication, understanding, and support of each other [54, 55]. Another study also mentioned that it can increase attention, affection, and wellbeing and improve quality of life [56]. Likewise, mindfulness exercises given to pregnant women with their husbands significantly affect their ability to act consciously (acting with awareness) and increase satisfaction and quality of relationships with partners [55]. During the study, husbands were more intense in participating in the training as they directly obtained information from health providers. Transparent information from these professionals makes fathers understand the right way and feel a deeper relationship with their wives and children and a closer relationship with the foetus [57].

Mindfulness for marital harmony has a very positive effect for mother, foetus, and husband. The mother showed behavioural changes, which are more patient and calmer in interacting and communicating with partners. The husband described that they were more concerned and alert to providing support, assistance, and involvement in the process of pregnancy and childbirth for their wives [51]. Meanwhile, the foetus also received a positive impact on growth and development [58]. In the maternity nursing area, mindfulness exercises for men improving interactions between father, mother, and foetus can further enhance harmonious relationships. Interactions are conducted by being alert to every moment of activity [57]. The father’s ability to feel the presence of the foetus can significantly increase the closeness of the emotional relationship between the father and the foetus [59], raise the man’s involvement in carrying out his role as a father [60], and increase the harmonious relationship of the father with a partner [61].

In a mindful state, the mind, emotions, and body sensations look at others with open and non-judgemental thought [61]. The quality benefits increase relationship attention, making the relationship healthier, reducing negative interaction, and increasing positive environment for the pregnancy [62]. Mindfulness nursing interventions that are performed individually affect partners to be more responsive because the principle of being fully present and non-judgemental causes a good perception of partner behaviour [40, 63]. This condition was also experienced by the husbands of the individual group in this current study. Even though these men did not get mindfulness training like the husbands of the paired group, they still had an impact on improving the value of the harmony of the husband-and-wife relationship that the father obtained.

### Strengths and limitations

The strengths of this study include (1) the intervention utilizing mHealth media, which allows respondents to access it without limitations of time and place (2) MIESRA mHealth, an application that provides crucial information during pregnancy, pre-test and posttest assessments for knowledge, and daily activities comprising instructional videos on mindfulness, audio materials, and diary notes, (3) MIESRA mHealth is easy to install, use, and comprehend, (4) detailed research methods were employed, and (5) statistical tests conducted by experts to ensure robust results. Additionally, the availability of this application can support the limited number and time constraints of health workers in providing antenatal health services. It enables monitoring of the psychological health of pregnant women and their partners, thereby enhancing the emotional bond between the mother and the foetus.

However, this study had several limitations, including (1) the participants were limited to a specific area. Therefore, conducting research in broader locations while considering beliefs and culture becomes essential; (2) MIESRA mHealth still had certain limitations. Researchers were unable to examine it in real-time to determine when participants engaged in mindfulness interventions and learning activities such as reading educational materials or watching educational videos. This aspect needs improvement in future work; (3) The interventions in this study were a single package; therefore, specific interventions were not conducted each week; (4) The generalization of research results is limited to pregnant women and partners without mental health disruptions. Further research is recommended for groups of patients diagnosed with mental illnesses. Overall, these limitations highlight the areas for improvement and suggest potential directions for future study.

## Conclusion

Nursing interventions to build emotional bonds between parents and foetuses based on mHealth can be a promising intervention for marital harmony during the perinatal period. The husband’s active involvement in the mindfulness exercise programme with his wife and interacting with the foetus yields psychological comfort and increases emotional bonds with his wife and foetus. Hence, the MIESRA mHealth can be one of solutions and suggestions to the Indonesia Government especially to increase the contribution of husbands in prenatal, antenatal, and postnatal care. We recommend the MIESRA mHealth as a nursing intervention that can implemented during pregnancy is integrated into the antenatal care programme to increase the marital satisfaction, foetal growth, and wellbeing in general.

## Supporting information

S1 Data MIESRA mHealth information and description. (PDF)

S2 Data (XLSX)

S1 File (DOCX)

## References

[1] Abduljalil K, Furness P, Johnson TN, Rostami-Hodjegan A, Soltani H. Anatomical, physiological and metabolic changes with gestational age during normal pregnancy: a database for parameters required in physiologically based pharmacokinetic modelling. Clin Pharmacokinet. 2012;51(6):365–96. doi: 10.2165/11597440-000000000-00000 . 22515555

[2] Hennekam S, Syed J, Ali F, Dumazert J-P. A multilevel perspective of the identity transition to motherhood. Gender, Work & Organization. 2019;26(7):915–33. doi: 10.1111/gwao.12334

[3] Soma-Pillay P, Nelson-Piercy C, Tolppanen H, Mebazaa A. Physiological changes in pregnancy. Cardiovasc J Afr. 2016;27(2):89–94. doi: 10.5830/CVJA-2016-021 . 27213856 PMC4928162

[4] Weissgerber TL, Wolfe LA. Physiological adaptation in early human pregnancy: adaptation to balance maternal-fetal demands. Appl Physiol Nutr Metab. 2006;31(1):1–11. doi: 10.1139/h05-003 . 16604136

[5] Rosnani R, Mediarti D. Overview of post-partum mother adaptation: A healthy lifestyle needs. The Journal of Palembang Nursing Studies. 2022;1(3):134–8. doi: 10.55048/jpns.v1i3.59

[6] Lonstein JS, Maguire J, Meinlschmidt G, Neumann ID. Emotion and mood adaptations in the peripartum female: complementary contributions of GABA and oxytocin. J Neuroendocrinol. 2014;26(10):649–64. doi: 10.1111/jne.12188 . 25074620 PMC5487494

[7] Moya J, Phillips L, Sanford J, Wooton M, Gregg A, Schuda L. A review of physiological and behavioral changes during pregnancy and lactation: potential exposure factors and data gaps. J Expo Sci Environ Epidemiol. 2014;24(5):449–58. Epub 20140115. doi: 10.1038/jes.2013.92 . 24424408

[8] O’Hara MW, Wisner KL. Perinatal mental illness: definition, description and aetiology. Best Pract Res Clin Obstet Gynaecol. 2014;28(1):3–12. Epub 2013/10/07. doi: 10.1016/j.bpobgyn.2013.09.002 . 24140480 PMC7077785

[9] Tambelli R, Ballarotto G, Trumello C, Babore A. Transition to motherhood: A Study on the Association between somatic symptoms during pregnancy and post-partum anxiety and depression symptoms. Int J Environ Res Public Health. 2022;19(19). Epub 20221007. doi: 10.3390/ijerph191912861 36232161 PMC9564583

[10] Miller ES, Hoxha D, Wisner KL, Gossett DR. The impact of perinatal depression on the evolution of anxiety and obsessive-compulsive symptoms. Arch Womens Ment Health. 2015;18(3):457–61. Epub 20141030. doi: 10.1007/s00737-014-0476-x 25355541 PMC7082147

[11] Yunitasari E, Pradanie R, Arifin H, Fajrianti D, Lee BO. Determinants of stunting prevention among mothers with children aged 6–24 months. Open Access Macedonian Journal of Medical Sciences. 2021;9:378–84. doi: 10.3889/oamjms.2021.6106

[12] Eastwood J, Ogbo FA, Hendry A, Noble J, Page A. The Impact of Antenatal Depression on Perinatal Outcomes in Australian Women. PLoS One. 2017;12(1):e0169907. Epub 20170117. doi: 10.1371/journal.pone.0169907 28095461 PMC5241141

[13] László KD, Liu XQ, Svensson T, Wikström AK, Li J, Olsen J, et al. Psychosocial stress related to the loss of a close relative the year before or during pregnancy and risk of preeclampsia. Hypertension. 2013;62(1):183–9. Epub 20130422. doi: 10.1161/HYPERTENSIONAHA.111.00550 . 23608651

[14] Kawanishi Y, Yoshioka E, Saijo Y, Itoh T, Miyamoto T, Sengoku K, et al. The relationship between prenatal psychological stress and placental abruption in Japan, The Japan Environment and Children’s Study (JECS). PLoS One. 2019;14(7):e0219379. Epub 20190708. doi: 10.1371/journal.pone.0219379 31283785 PMC6613679

[15] Beeghly M, Partridge T, Tronick E, Muzik M, Rahimian Mashhadi M, Boeve JL, et al. Associations between early maternal depressive symptom trajectories and toddlers’ felt security at 18 months: are boys and girls at differential risk? Infant mental health journal. 2017;38(1):53–67. Epub 2017/01/02. doi: 10.1002/imhj.21617 . 28042661 PMC5225085

[16] Henriksen RE, Thuen F. Marital quality and stress in pregnancy predict the risk of infectious disease in the offspring: The Norwegian mother and child cohort study. PloS one. 2015;10(9):e0137304–e. doi: 10.1371/journal.pone.0137304 . 26422017 PMC4589358

[17] Zaheri F, Dolatian M, Shariati M, Simbar M, Ebadi A, Azghadi SBH. Effective factors in marital satisfaction in perspective of iranian women and men: A systematic review. Electron Physician. 2016;8(12):3369–77. doi: 10.19082/3369 . 28163850 PMC5279968

[18] Ammaniti M, Trentini C, Menozzi F, Tambelli R. Transition to parenthood: studies of intersubjectivity in mothers and fathers. Early Parenting and Prevention of Disorder: Routledge; 2018. p. 129–64.

[19] Trentini C, Pagani M, Lauriola M, Tambelli R. Neural responses to infant emotions and emotional self-awareness in mothers and fathers during pregnancy. Int J Environ Res Public Health. 2020;17(9). Epub 20200509. doi: 10.3390/ijerph17093314 32397541 PMC7246792

[20] Mercer RT. Becoming a mother versus maternal role attainment. J Nurs Scholarsh. 2004;36(3):226–32. doi: 10.1111/j.1547-5069.2004.04042.x . 15495491

[21] Rafii F, Alinejad-Naeini M, Peyrovi H. Maternal role attainment in mothers with term neonate: A hybrid concept analysis. Iranian journal of nursing and midwifery research. 2020;25(4):304–13. doi: 10.4103/ijnmr.IJNMR_201_19 . 33014742 PMC7494165

[22] Taghani R, Ashrafizaveh A, Ghanbari Soodkhori M, Azmoude E, Tatari M. Marital satisfaction and its associated factors at reproductive age women referred to health centers. Journal of education and health promotion. 2019;8:133-. doi: 10.4103/jehp.jehp_172_18 . 31463318 PMC6691613

[23] Sevinç M, Garip ES. A study of parents’ child raising styles and marital harmony. Procedia—Social and Behavioral Sciences. 2010;2(2):1648–53. doi: 10.1016/j.sbspro.2010.03.252

[24] Kamp Dush CM, Taylor MG, Kroeger RA. Marital happiness and psychological well-being across the life course. Family relations. 2008;57(2):211–26. doi: 10.1111/j.1741-3729.2008.00495.x . 23667284 PMC3650717

[25] Stapleton LRT, Schetter CD, Westling E, Rini C, Glynn LM, Hobel CJ, et al. Perceived partner support in pregnancy predicts lower maternal and infant distress. J Fam Psychol. 2012;26(3):453–63. doi: 10.1037/a0028332 . 22662772 PMC3992993

[26] Yalcintas S, Pike A. Co-parenting and marital satisfaction predict maternal internalizing problems when expecting a second child. Psychological Studies. 2021;66(2):212–9. doi: 10.1007/s12646-021-00620-z 34341619 PMC8319188

[27] Mediarti D, Rosnani R, Sukartini T, Arifin H, Kurniawati Y. Coverage and factors associated with complete polio vaccination among Indonesian children aged 0–18 months. Children and Youth Services Review. 2020;118:105399-. doi: 10.1016/j.childyouth.2020.105399

[28] Kusnanto K, Arifin H, Kurniawati Y. Determinant of BCG vaccine coverage among Indonesian children aged 0–2 months. Children and Youth Services Review. 2020;116. doi: 10.1016/j.childyouth.2020.105238

[29] Lewis S, Lee A, Simkhada P. The role of husbands in maternal health and safe childbirth in rural Nepal: a qualitative study. BMC Pregnancy and Childbirth. 2015;15(1):162. doi: 10.1186/s12884-015-0599-8 26239123 PMC4523911

[30] Mercer RT. Nursing support of the process of becoming a mother. J Obstet Gynecol Neonatal Nurs. 2006;35(5):649–51. doi: 10.1111/j.1552-6909.2006.00086.x . 16958722

[31] Yekta FF, Yaghoubi H, Ghomian S, Gholami Fesharaki M. Mediators for effect of mindfulness in promoting marital satisfaction: Modeling structural equations in an experimental study. Iran J Psychiatry. 2022;17(1):72–83. doi: 10.18502/ijps.v17i1.8051 35480126 PMC8994843

[32] Atkinson B. Mindfulness training and the cultivation of secure, satisfying couple relationships. Couple and Family Psychology: Research and Practice. 2013;2:73. doi: 10.1037/cfp0000002

[33] Duncan LG, Bardacke N. Mindfulness-based childbirth and parenting education: promoting family mindfulness during the perinatal period. Journal of child and family studies. 2010;19(2):190–202. Epub 2009/10/10. doi: 10.1007/s10826-009-9313-7 . 20339571 PMC2837157

[34] Duncan LG, Shaddix C. Mindfulness-based childbirth and parenting (mbcp): Innovation in birth preparation to support healthy, happy families. Int J Birth Parent Educ. 2015;2(2):30–3. . 29051821 PMC5645068

[35] Burpee LC, Langer EJ. Mindfulness and marital satisfaction. Journal of Adult Development. 2005;12(1):43–51. doi: 10.1007/s10804-005-1281-6

[36] Klankhajhon S, Sthien A. A narrative review of physical activity and exercise during pregnancy: Nurse’s role. The Journal of Palembang Nursing Studies. 2022;1(2):49–60. doi: 10.55048/jpns.v1i2.16

[37] Liu Y, Wang X. Application of smart mobile medical services in maternal health care management. Contrast Media Mol Imaging. 2021;2021:6249736-. doi: 10.1155/2021/6249736 . 34949971 PMC8674054

[38] Abedi Shargh N, Bakhshani NM, Mohebbi MD, Mahmudian K, Ahovan M, Mokhtari M, et al. The effectiveness of mindfulness-based cognitive group therapy on marital satisfaction and general health in woman with infertility. Global journal of health science. 2015;8(3):230–5. doi: 10.5539/gjhs.v8n3p230 . 26493418 PMC4803966

[39] Gharibboluk M, Hosseinzadeh S. The effectiveness of mindfulness training on quality of perceptual marital relationship and psychological well-being of women with addicted wife. Revista Romaneasca pentru Educatie Multidimensionala. 2018;10(1.Suppl):34–46. doi: 10.18662/rrem/35

[40] Kappen G, Karremans JC, Burk WJ. Effects of a short online mindfulness intervention on relationship satisfaction and partner acceptance: The moderating role of trait mindfulness. Mindfulness. 2019;10(10):2186–99.

[41] Harris AD, McGregor JC, Perencevich EN, Furuno JP, Zhu J, Peterson DE, et al. The use and interpretation of quasi-experimental studies in medical informatics. Journal of the American Medical Informatics Association. 2006;13(1):16–23. doi: 10.1197/jamia.M1749 16221933 PMC1380192

[42] Kusnanto K, Arifin H, Pradipta RO, Gusmaniarti G, Kuswanto H, Setiawan A, et al. Resilience-based Islamic program as a promising intervention on diabetes fatigue and health-related quality of life. PLOS ONE. 2022;17(9):e0273675. doi: 10.1371/journal.pone.0273675 36048792 PMC9436096

[43] Faul F, Erdfelder E, Buchner A, Lang AG. Statistical power analyses using G*Power 3.1: tests for correlation and regression analyses. Behav Res Methods. 2009;41(4):1149–60. doi: 10.3758/BRM.41.4.1149 . 19897823

[44] Faul F, Erdfelder E, Lang AG, Buchner A. G*Power 3: a flexible statistical power analysis program for the social, behavioral, and biomedical sciences. Behav Res Methods. 2007;39(2):175–91. doi: 10.3758/bf03193146 . 17695343

[45] Ismail RI. Kuesioner kesesuaian hubungan suami istri (KHSI). Depok, Indonesia: Universitas Indonesia, 2003.

[46] Ismail RI, Anindyajati G, Diatri H, Elvira SD. Reliability testing of screening instruments for antenatal depression and associated risk factors in urban primary care. Advanced Science Letters. 2017;23(4):3445–7. doi: 10.1166/asl.2017.9124

[47] Nielsen J. Reliability of severity estimates for usability problems found by heuristic evaluation. Posters and Short Talks of the 1992 SIGCHI Conference on Human Factors in Computing Systems; Monterey, California: Association for Computing Machinery; 1992. p. 129–30.

[48] Sharfina Z, Santoso HB, editors. An Indonesian adaptation of the System Usability Scale (SUS). 2016 International Conference on Advanced Computer Science and Information Systems (ICACSIS); 2016 15–16 Oct. 2016.

[49] Lauderdale S. Book Review: Biostatistics: A methodology for the health sciences, 2nd Edition. Annals of Pharmacotherapy. 2005;39(3):576–7. doi: 10.1345/aph.1E470

[50] Ghisletta P, Spini D. An introduction to generalized estimating equations and an application to assess selectivity effects in a longitudinal study on very old individuals. Journal of Educational and Behavioral Statistics. 2004;29(4):421–37. doi: 10.3102/10769986029004421

[51] Lönnberg G, Nissen E, Niemi M. What is learned from mindfulness based childbirth and parenting education?—Participants’ experiences. BMC Pregnancy Childbirth. 2018;18(1):466. Epub 20181203. doi: 10.1186/s12884-018-2098-1 30509218 PMC6276167

[52] Nursalam N, Sukartini T, Arifin H, Pradipta RO, Mafula D, Ubudiyah M. Determinants of the discriminatory behavior experienced by people living with hiv in Indonesia: A Cross-sectional study of the demographic health survey. The Open AIDS Journal. 2021;15(1):1–9. doi: 10.2174/1874613602115010001

[53] Stanton SCE, Chan APS, Gazder T. Mindfulness, perceived partner responsiveness, and relationship quality: A dyadic longitudinal mediation model. Journal of Social and Personal Relationships. 2021;38(11):3310–32. doi: 10.1177/02654075211030327

[54] Hinckley E, Favorite T, Nickelsen T, King A. Effects on marital satisfaction and resting state functional connectivity in default mode network (DMN): A pilot study of a mindfulness- and loving-kindness meditation- based group couples relationship enhancement (MBRE) intervention for military veterans and spouses. Biological Psychiatry. 2020;87(9, Supplement):S390. doi: 10.1016/j.biopsych.2020.02.998

[55] Xie J, Zhou D, Tan Y. Relationship between mindfulness and general health among couples in Mainland China: A crossover perspective. Social Science & Medicine. 2021;281:114095. doi: 10.1016/j.socscimed.2021.114095 34130075

[56] Winter F, Steffan A, Warth M, Ditzen B, Aguilar-Raab C. Mindfulness-based couple interventions: A systematic literature review. Family Process. 2021;60(3):694–711. doi: 10.1111/famp.12683 34114656

[57] Gambrel LE, Piercy FP. Mindfulness-based relationship education for couples expecting their first child—Part 1: A Randomized Mixed-Methods Program Evaluation. Journal of Marital and Family Therapy. 2015;41(1):5–24. doi: 10.1111/jmft.12066 24433518

[58] Chan KP. Prenatal meditation influences infant behaviors. Infant Behavior and Development. 2014;37(4):556–61. doi: 10.1016/j.infbeh.2014.06.011 25063985

[59] Vreeswijk CM, Maas AJ, Rijk CH, van Bakel HJ. Fathers’ experiences during pregnancy: Paternal prenatal attachment and representations of the fetus. Psychology of Men & Masculinity. 2014;15(2):129.

[60] Brandon AR, Pitts S, Denton WH, Stringer CA, Evans H. A history of the theory of prenatal attachment. Journal of prenatal & perinatal psychology & health: APPPAH. 2009;23(4):201. 21533008 PMC3083029

[61] Simonelli A, Parolin M, Sacchi C, De Palo F, Vieno A. The role of father involvement and marital satisfaction in the development of family interactive abilities: A multilevel approach. Frontiers in psychology. 2016;7:1725. doi: 10.3389/fpsyg.2016.01725 27872601 PMC5098289

[62] Atkinson BJ. Mindfulness training and the cultivation of secure, satisfying couple relationships. Couple and Family Psychology: Research and Practice. 2013;2(2):73.

[63] Adair KC, Boulton AJ, Algoe SB. The effect of mindfulness on relationship satisfaction via perceived responsiveness: Findings from a dyadic study of heterosexual romantic partners. Mindfulness. 2018;9(2):597–609.

