# Supplementary figures and images for: MIESRA mHealth: Marital satisfaction during pregnancy
Source: PLoS One. 2023 Aug 24;18(8):e0289061. doi: 10.1371/journal.pone.0289061 (PMC10449122; doi:10.1371/journal.pone.0289061)

1.

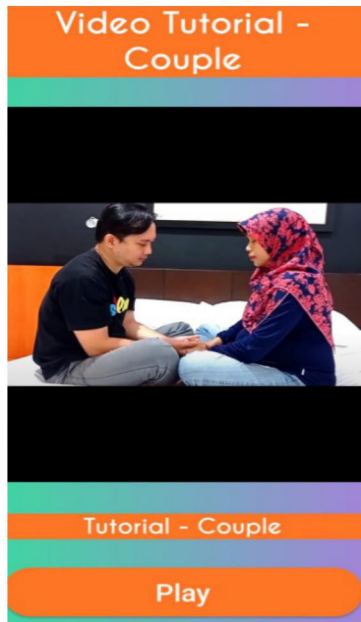

(a)

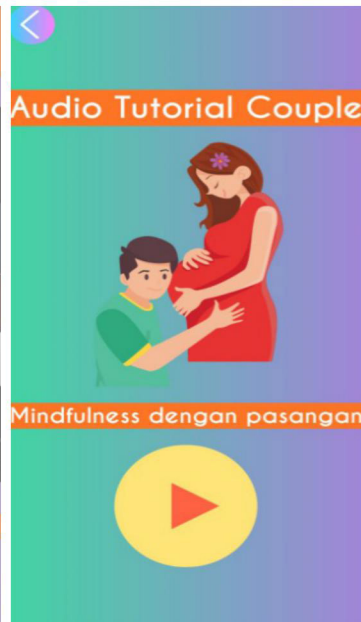

(b)

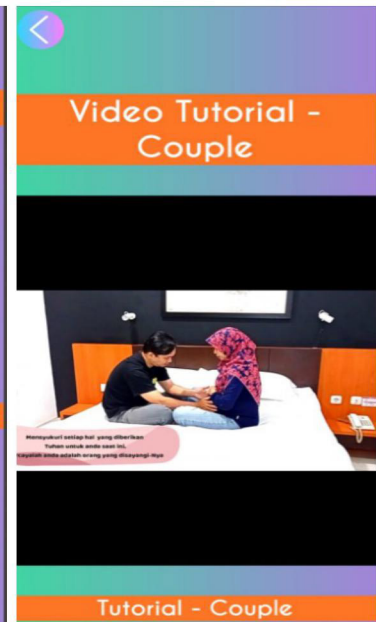

(c)

2.

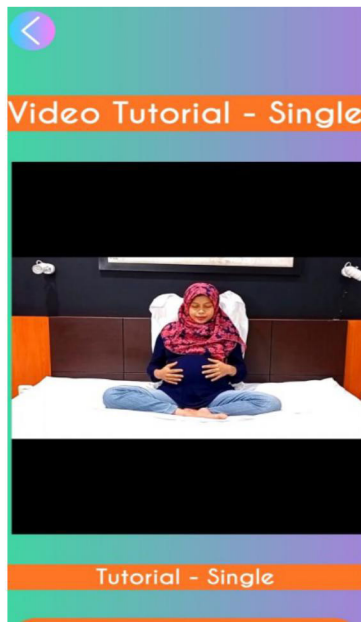

(a)

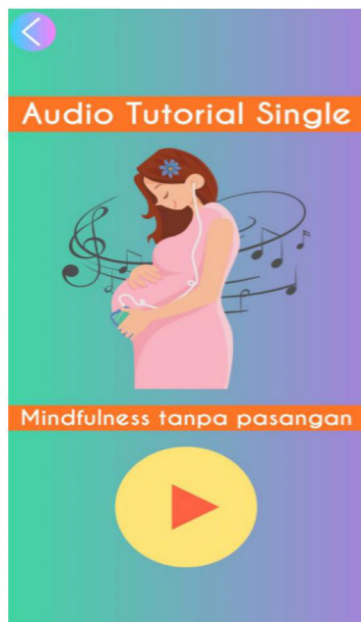

(b)

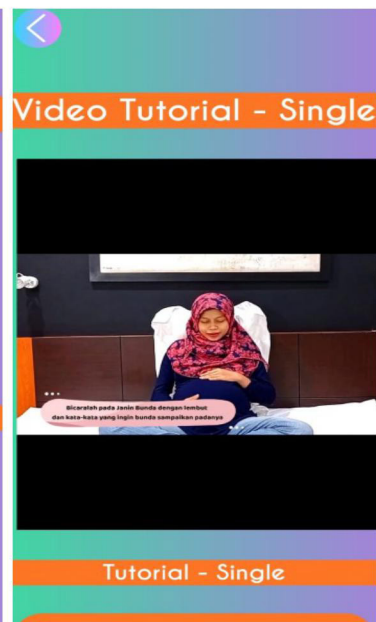

(c)

3.

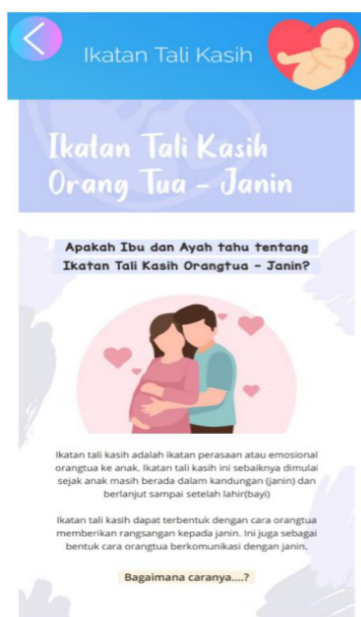

(a)

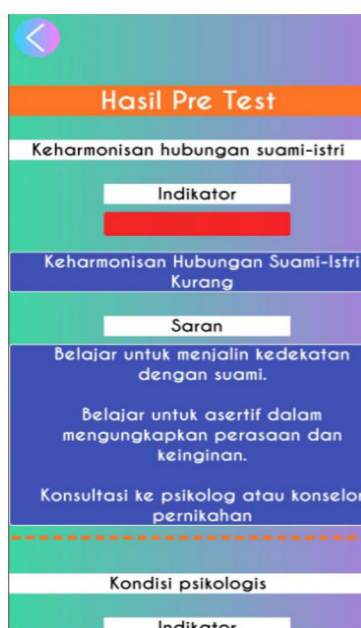

(b)

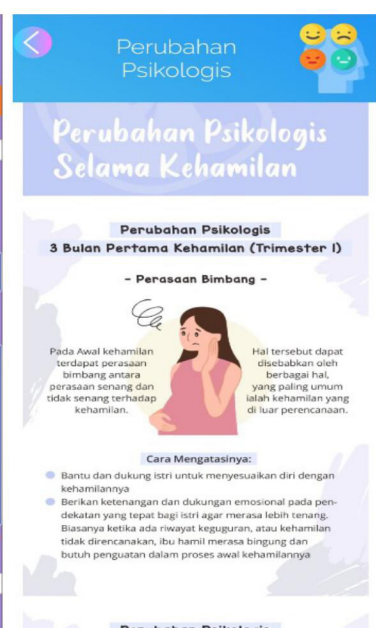

(c)

Supplement: S1 Data — (PDF) [file pone.0289061.s001.pdf]
