# Supplementary material for: MIESRA mHealth: Marital satisfaction during pregnancy
Source: PLoS One. 2023 Aug 24;18(8):e0289061. doi: 10.1371/journal.pone.0289061 (PMC10449122; doi:10.1371/journal.pone.0289061)
Supplement: S1 File — (DOCX) [file pone.0289061.s003.docx]

**Supplementary 1**

In this supplement, there is information and descriptions related to the MIESRA mHealth. Figure S1 displays the MIESRA mHealth for a couple group, including a video (1a & 1c) demonstrating husband-wife mindfulness and providing guidance on correct mindfulness practice, as well as an audio tutorial (1b) for mindfulness. Figure S1 also shows the MIESRA mHealth for single groups, featuring a video (2a & 2c) with a guide for mothers to practice mindfulness independently, along with accompanying text, and an audio tutorial (2b) for the single group. Additionally, Figure S1 provides information regarding education (3a-3c) and showcases the pre-post-test feature, enabling immediate result visualization.
