# Supplementary material for: MIESRA mHealth: Marital satisfaction during pregnancy
Source: PLoS One. 2023 Aug 24;18(8):e0289061. doi: 10.1371/journal.pone.0289061 (PMC10449122; doi:10.1371/journal.pone.0289061)
Supplement: Attachment — Submitted filename: Response to Reviewer - PlosOne.docx [file pone.0289061.s004.docx]

**Response to Editor and Reviewers**

**To Editor**

1. Please ensure that your manuscript meets PLOS ONE's style requirements, including those for file naming.

**Response**: Thank you, we have followed the guidelines.

2. In your Data Availability statement, you have not specified where the minimal data set underlying the results described in your manuscript can be found. PLOS defines a study's minimal data set as the underlying data used to reach the conclusions drawn in the manuscript and any additional data required to replicate the reported study findings in their entirety.

**Response**: Thank you, we have uploaded the raw data of this study.

“All data relevant to the study are included in the article and its Supporting Information files”

3. Please include a caption for figure 2

**Response**: Thank you, we have added the caption for figure 2.

4. We note that Figure S1 and S2 includes an image of a patient/participant in the study.
   As per the PLOS ONE policy (<http://journals.plos.org/plosone/s/submission-guidelines#loc-human-subjects-research>) on papers that include identifying, or potentially identifying, information, the individual(s) or parent(s)/guardian(s) must be informed of the terms of the PLOS open-access (CC-BY) license and provide specific permission for publication of these details under the terms of this license. Please download the Consent Form for Publication in a PLOS Journal (<http://journals.plos.org/plosone/s/file?id=8ce6/plos-consent-form-english.pdf>). The signed consent form should not be submitted with the manuscript, but should be securely filed in the individual's case notes. Please amend the methods section and ethics statement of the manuscript to explicitly state that the patient/participant has provided consent for publication: “The individual in this manuscript has given written informed consent (as outlined in PLOS consent form) to publish these case details”.

**Response**: Thank you for the correction. We have filled the consent form for publication and mentioned the statement in the ethical consideration's part.

5. Please include captions for your Supporting Information files at the end of your manuscript, and update any in-text citations to match accordingly. Please see our Supporting Information guidelines for more information: <http://journals.plos.org/plosone/s/supporting-information>.

**Response**: Thank you, we have added the supporting information.

6. Please review your reference list to ensure that it is complete and correct

**Response**: Revised. Thank you,

**Reviewer 1**

1. Very interesting and good topic that had been discussed. The importance of the study had been highlighted clearly at the begining of the writing.

**Response**: Thank you,

2. How the implementation of the treatments for all three types of group had been discussed. The inclusion criteria for the sample also clearly stated. However, would like to know is the information of income being asked to the pregnant women? If no, could the author provide justification.

**Response**: Thank you for your question. We did not ask pregnant women about their income because, for our participants, the primary source of income was their husbands. Therefore, we refrain from inquiring about income from pregnant women.

3. Then, the experiment being done for 4 weeks, what would be the details of the content for each week? Would be good if can add it in writing.

**Response**: Thank you for the question. In this study, we are unable to provide detailed information about the intervention content for each week because the interventions in our study were implemented as a single package. We have included the details of the study procedure in the study procedure section. However, this is a valuable suggestion, so we have added this information to the study limitations.

4. Is it standardized with the other group regarding the content exposed for each of the weeks?

**Response**: Yes, the contents were standardized.

5. Lastly suggest to highlight the strength of the MIESRA in the strenght and limitation.

**Response**: Thank you for the suggestion. We have revised the strengths and limitation of this study.

“The strengths of this study include (1) the intervention utilizing mHealth media, which allows respondents to access it without limitations of time and place (2) MIESRA mHealth, an application that provides crucial information during pregnancy, pre-test and posttest assessments for knowledge, and daily activities comprising instructional videos on mindfulness, audio materials, and diary notes, (3) MIESRA mHealth is easy to install, use, and comprehend, (4) detailed research methods were employed, and (5) statistical tests conducted by experts to ensure robust results. Additionally, the availability of this application can support the limited number and time constraints of health workers in providing antenatal health services. It enables monitoring of the psychological health of pregnant women and their partners, thereby enhancing the emotional bond between the mother and the foetus.

However, this study had several limitations, including (1) the participants were limited to a specific area. Therefore, conducting research in broader locations while considering beliefs and culture becomes essential; (2) MIESRA mHealth still had certain limitations. Researchers were unable to examine it in real-time to determine when participants engaged in mindfulness interventions and learning activities such as reading educational materials or watching educational videos. This aspect needs improvement in future work; (3) The interventions in this study were a single package; therefore, specific interventions were not conducted each week; (4) The generalization of research results is limited to pregnant women and partners without mental health disruptions. Further research is recommended for groups of patients diagnosed with mental illnesses. Overall, these limitations highlight the areas for improvement and suggest potential directions for future study.”

Reviewer 2

1. Thank you for the opportunity to review this study. I think the topic is very important, as has been well pointed out by the authors. Overall, the paper was well written and there are only a few points that could be revised.

**Response**: Thank you,

2. Specifically, in reviewing the existing literature, it would be useful to look more into the transition that women go through. I cite some studies as examples:

- Tambelli, R., Ballarotto, G., Trumello, C., & Babore, A. (2022). Transition to Motherhood: A Study on the Association between Somatic Symptoms during Pregnancy and Post-Partum Anxiety and Depression Symptoms. International Journal of Environmental Research and Public Health, 19(19), 12861.
- Hennekam, S., Syed, J., Ali, F., & Dumazert, J. P. (2019). A multilevel perspective of the identity transition to motherhood. Gender, Work & Organization, 26(7), 915-933.
- Trentini, C., Pagani, M., Lauriola, M., & Tambelli, R. (2020). Neural responses to infant emotions and emotional self-awareness in mothers and fathers during pregnancy. International journal of environmental research and public health, 17(9), 3314.
- Ammaniti, M., Trentini, C., Menozzi, F., & Tambelli, R. (2014). Transition to parenthood: Studies of intersubjectivity in mothers and fathers.
- Duarte-Guterman, P., Leuner, B., & Galea, L. A. (2019). The long and short term effects of motherhood on the brain. Frontiers in neuroendocrinology, 53, 100740.

**Response**: Thank you for your valuable suggestion, we have provided some information about transition women. We have elaborated this information in the introduction.

3. In addition, it would be helpful if the authors provided more information regarding the content of the application.

**Response**: Thank you for the comment. The information regarding MIESRA mHealth contents were presented in the table 1.

4. Finally, the authors are invited to highlight strengths and weaknesses of MIESRA, but especially the future directions that the study's findings imply

**Response**: Thank you for the suggestion. We have revised the strengths and limitation of this study.

“The strengths of this study include (1) the intervention utilizing mHealth media, which allows respondents to access it without limitations of time and place (2) MIESRA mHealth, an application that provides crucial information during pregnancy, pre-test and posttest assessments for knowledge, and daily activities comprising instructional videos on mindfulness, audio materials, and diary notes, (3) MIESRA mHealth is easy to install, use, and comprehend, (4) detailed research methods were employed, and (5) statistical tests conducted by experts to ensure robust results. Additionally, the availability of this application can support the limited number and time constraints of health workers in providing antenatal health services. It enables monitoring of the psychological health of pregnant women and their partners, thereby enhancing the emotional bond between the mother and the foetus.

However, this study had several limitations, including (1) the participants were limited to a specific area. Therefore, conducting research in broader locations while considering beliefs and culture becomes essential; (2) MIESRA mHealth still had certain limitations. Researchers were unable to examine it in real-time to determine when participants engaged in mindfulness interventions and learning activities such as reading educational materials or watching educational videos. This aspect needs improvement in future work; (3) The interventions in this study were a single package; therefore, specific interventions were not conducted each week; (4) The generalization of research results is limited to pregnant women and partners without mental health disruptions. Further research is recommended for groups of patients diagnosed with mental illnesses. Overall, these limitations highlight the areas for improvement and suggest potential directions for future study.”
